# Application and limitation of a biological clock-based method for estimating time of death in forensic practices

**DOI:** 10.1038/s41598-023-33328-3

**Published:** 2023-04-13

**Authors:** Akihiko Kimura, Yuko Ishida, Mizuho Nosaka, Akiko Ishigami, Hiroki Yamamoto, Yumi Kuninaka, Satoshi Hata, Mitsunori Ozaki, Toshikazu Kondo

**Affiliations:** 1 Department of Forensic Medicine, Wakayama Medical University, 811-1 Kimiidera, Wakayama, 641-8509 Japan; 2 Department of Cardiovascular Medicine, Kinan Hospital, Wakayama, Japan; 3 Department of Neurological Surgery, National Hospital Organization Minami Wakayama Medical Center, Wakayama, Japan

**Keywords:** Biomarkers, Medical research

## Abstract

Estimating time of death is one of the most important problems in forensics. Here, we evaluated the applicability, limitations and reliability of the developed biological clock-based method. We analyzed the expression of the clock genes, BMAL1 and NR1D1, in 318 dead hearts with defined time of death by real-time RT-PCR. For estimating the time of death, we chose two parameters, the NR1D1/BMAL1 ratio and BMAL1/NR1D1 ratio for morning and evening deaths, respectively. The NR1D1/BMAL1 ratio was significantly higher in morning deaths and the BMAL1/NR1D1 ratio was significantly higher in evening deaths. Sex, age, postmortem interval, and most causes of death had no significant effect on the two parameters, except for infants and the elderly, and severe brain injury. Although our method may not work in all cases, our method is useful for forensic practice in that it complements classical methods that are strongly influenced by the environment in which the corpse is placed. However, this method should be applied with caution in infants, the elderly, and patients with severe brain injury.

## Introduction

Estimating the time of death, which is often extremely difficult, is one of the most important tasks in forensic practice. To date, numerous methods for estimating the time of death have been developed^1,2^. Over the last decade, various innovative techniques, such as tissue nano mechanics^3^, mass spectrometry-based quantitative proteomics^4^, analysis of oral microbiota community^5^ and micro-RNA analysis^6^, have been introduced to estimate the postmortem interval, bringing substantial progress into this field. However, most of these methods estimate the time since death, but not estimate the time of death. The current method for estimating the time of death remains unsatisfactory.

Advances in chronobiology have brought about great impacts and progress in various medical fields, such as chronopharmacology, chronotherapy and sleep disorder therapy^7–14^. Chronobiology can contribute to forensic medicine, especially in the estimation of the time of death. However, the forensic application of chronobiology is quite limited. To our knowledge, there is currently only one report of the application of chronobiology to forensic investigation, in which the time of death was estimated based on the melatonin concentration in pineal body, serum and urine^15^. Therefore, we tried to apply the biological clock to the estimation of the time of death. In 2011, we reported the first forensic application of chronobiology in the estimation of the time of death using a mouse model and applied the method to a few autopsy cases^16^. In our previous report, we used two main oscillator genes, brain and muscle aryl hydrocarbon receptor nuclear translocator-like 1 (*BMAL1* or *ARNTL*) and nuclear receptor subfamily 1 group D member 1 (Rev-Erbα, *NR1D1*), in the circadian clock system to read the biological clock in the kidneys, livers and hearts. Since these two clock genes oscillate in opposite phases^17,18^, the *NR1D1*/*BMAL1* ratio amplifies the circadian oscillation of each gene expression^16^. We demonstrated the applicability of our method in forensic practice, but we could not clarify the reliability and limitations of the method, because only a limited number of autopsy cases were examined.

Since its development, we have applied the method to our routine practice of estimating the time of death in autopsy cases. In this study, we evaluated our method based on the results of its application to 318 autopsy cases with known times of death in our department. We show the practical applicability and limitations of our method, which estimates the time of death based on the biological clock.

## Results

### The pattern of clock gene expression in the hearts of autopsy cases

**Figure 1:**
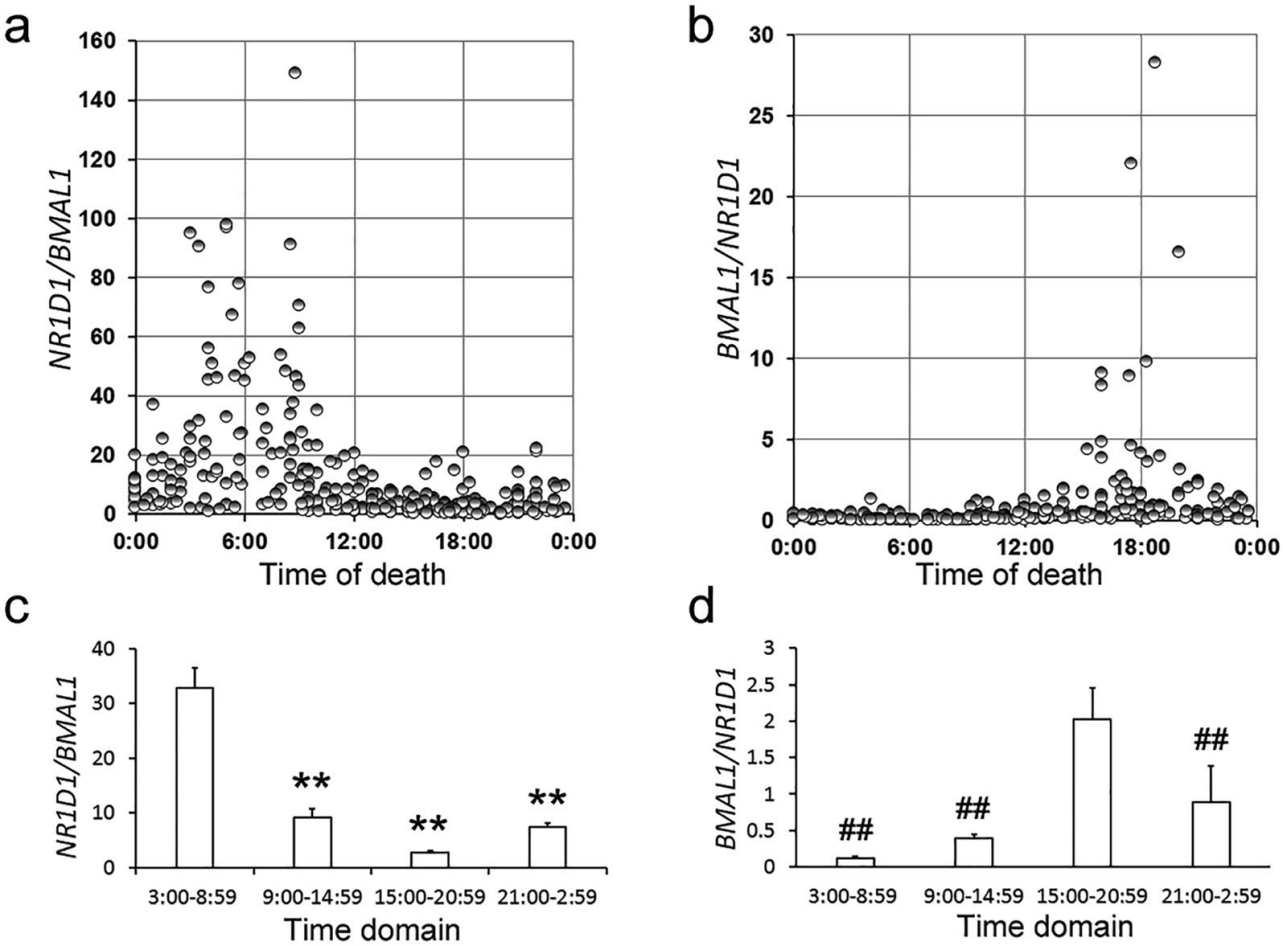
Temporal pattern of the *N/B* and *B/N* ratios in autopsy cases. The *N/B* (**a**) and *B/N* (**b**) ratios were plotted against the time of death. The autopsy cases were divided into four-time domains, and the *N/B* (**c**) and *B/N* (**d**) ratios in each time domain were examined by multiple comparison tests. **p < 0.01, 3:00–8:59 time domain versus other time domains; ^##^*p* < 0.01, 15:00–20:59 time domain versus other time domains.

The *NR1D1/BMAL1* (*N/B*) and *BMAL1/NR1D1* (*B/N*) ratios were plotted against the time of death, resulting in clear peaks around 6:00 and 18:00, respectively (Fig. 1a and b), indicating that clock gene expression can be precisely detected even in dead bodies.

Figure 1c and d show the mean values of the *N/B* and *B/N* ratio in the four-time domains (morning, 3:00–8:59, noon, 9:00–14:59, evening, 15:00–20:59 and night, 21:00–2:59). The *N/B* and *B/N* ratios were significantly higher in the morning and evening than in the other time domains, respectively, which confirms that these ratios are suitable parameters for estimating the time of death. However, in some autopsy cases, the *N/B* and *B/N* ratios exhibited very low values in the morning and evening, respectively (Fig. 1a and b), suggesting that some factors affected these parameters.

### Evaluation of the factors affecting the biological clock in the deceased

**Figure 2:**
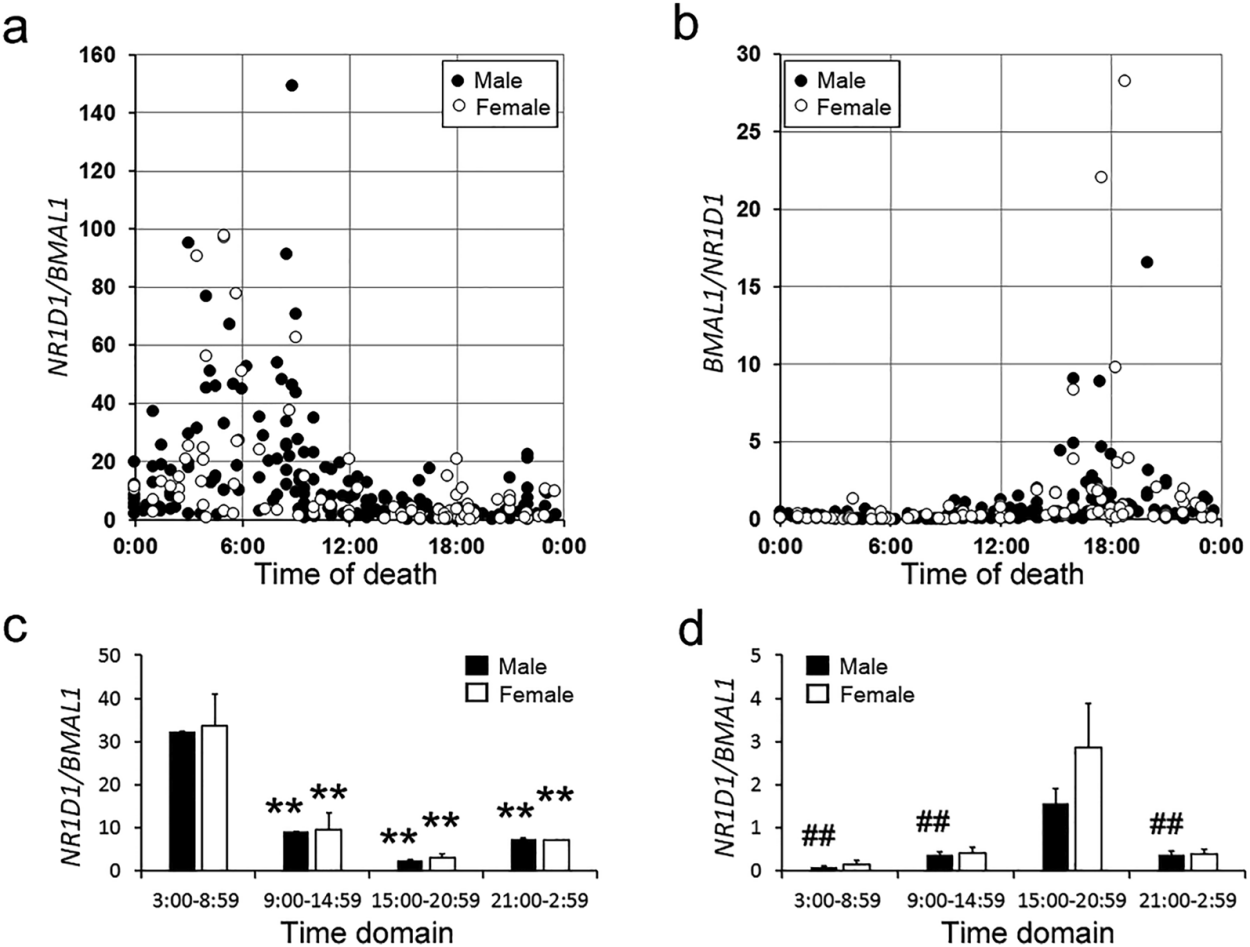
Assessment of the effect of gender on the *N/B* (**a**) and *B/N* (**b**) ratios in the hearts of the deceased. The *N/B* and *B/N* ratios in males (closed circles, n = 224) and females (open circles, n = 94) were plotted against the time of death. The *N/B* (**c**) and *B/N* (**d**) ratios in four-time domains were examined in males (solid columns) and females (open columns) by multiple comparison tests. **p < 0.01, 3:00–8:59 time domain versus other time domains; ^##^*p* < 0.01, 15:00–20:59 time domain versus other time domains.

We next examined the factors affecting the ratios in the deceased. First, we examined gender differences in the temporal pattern of the ratios (male, n = 224; female, n = 94). Both genders showed a similar temporal pattern of the *N/B* (Fig. 2a) and *B/N* (Fig. 2b) ratios. The N*/B* (Fig. 2c) and *B/N* (Fig. 2d) ratios in deceased males were significantly higher in the morning and evening, respectively, which was similar to the results of total cases (Fig. 1c and d). On the other hand, the *N/B* ratio in deceased females (Fig. 2c) was significantly higher in the morning, which is similar to the results in deceased males, whereas the *B/N* ratio was higher in the evening than in other time domains, but the difference was not statistically significant. (Fig. 2d).

**Figure 3:**
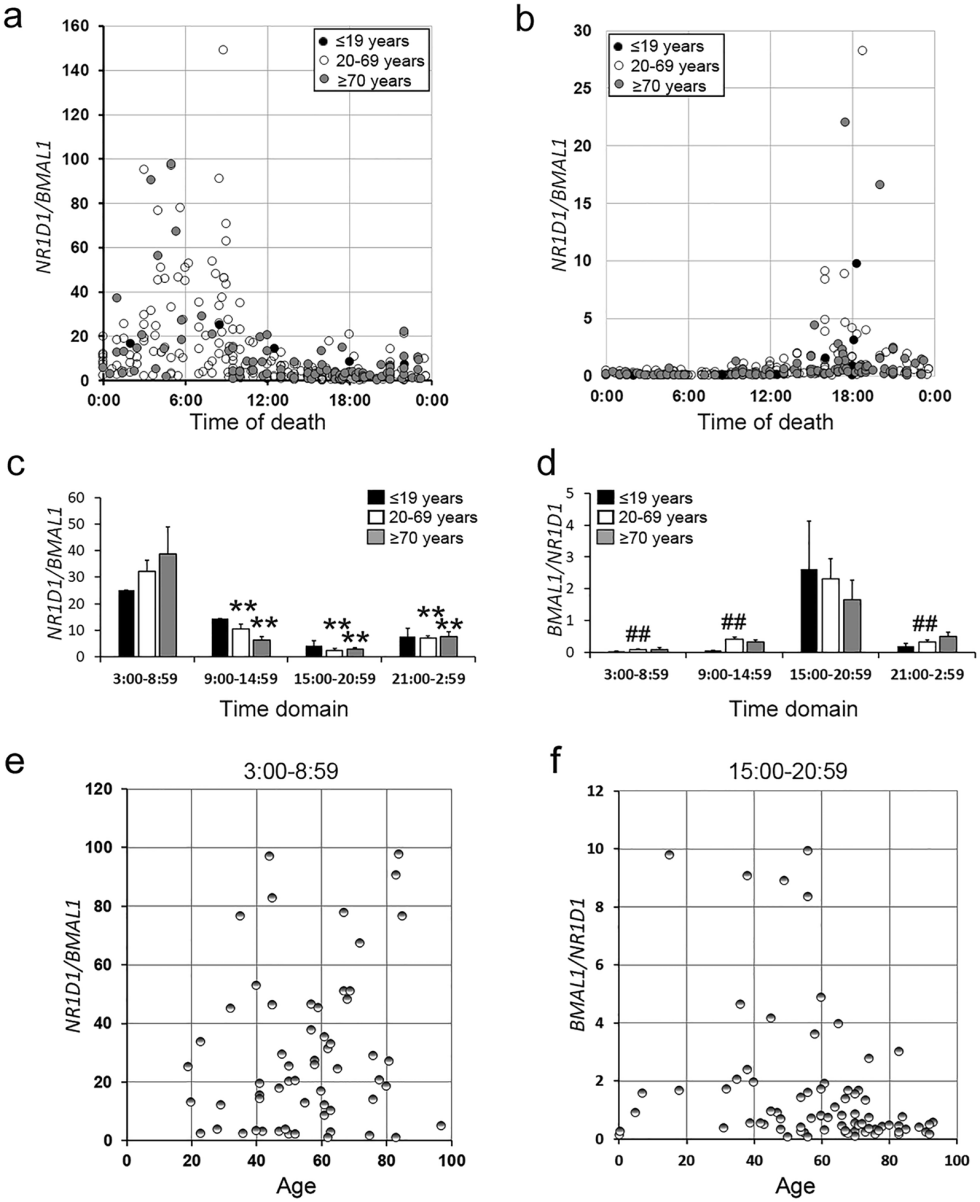
Assessment of the effect of age on the *N/B* (**a**) and *B/N* (**b**) ratios in the hearts of the deceased. The autopsy cases were divided into three age groups: ≤ 19 years (closed circles, n = 13), 20–69 years, (open circles, n = 200), and ≥ 70 years (gray circles, n = 105). The *N/B* (**c**) and *B/N* (**d**) ratios in four-time domains were examined in the ≤ 19 years (solid columns), 20–69 years (open columns), and ≥ 70 years (gray columns) groups by multiple comparison tests. The *N/B* ratios in the 3:00–8:59 time domain (**e**) and the *B/N* ratio in the 15:00–20:59 time domain (**f**) were plotted against age. **p < 0.01, 3:00–8:59 time domain versus other time domains; ^##^*p* < 0.01, 15:00–20:59 time domain versus other time domains.

We divided the cases into three age groups (≤ 19 years, n = 13; 20–69 years, n = 200; ≥ 70 years, n = 105). All age groups showed similar temporal patterns (Fig. 3a–d). The N/B ratio in the morning was significantly higher than those in the three other time domains in the 20–69 and ≥ 70 years groups (Fig. 3c). The B/N ratio in the evening was higher than those in the three other time domains only in the 20–69 years group (Fig. 3d). In contrast, the temporal pattern of the N/B ratio in the morning (3:00–8:59) and that of the B/N ratio in the evening (15:00–20:59) did not significantly differ from those in other time domains in the ≤ 19 years group (Fig. 3c and d). The N/B ratio in the morning and the B/N ratio in the evening were plotted against age; the results showed that the N/B and B/N ratios are independent of age (Fig. 3e and f). However, the case number in young and high-age groups was small. Therefore, more cases must be used for statistical analysis of these groups.

**Figure 4:**
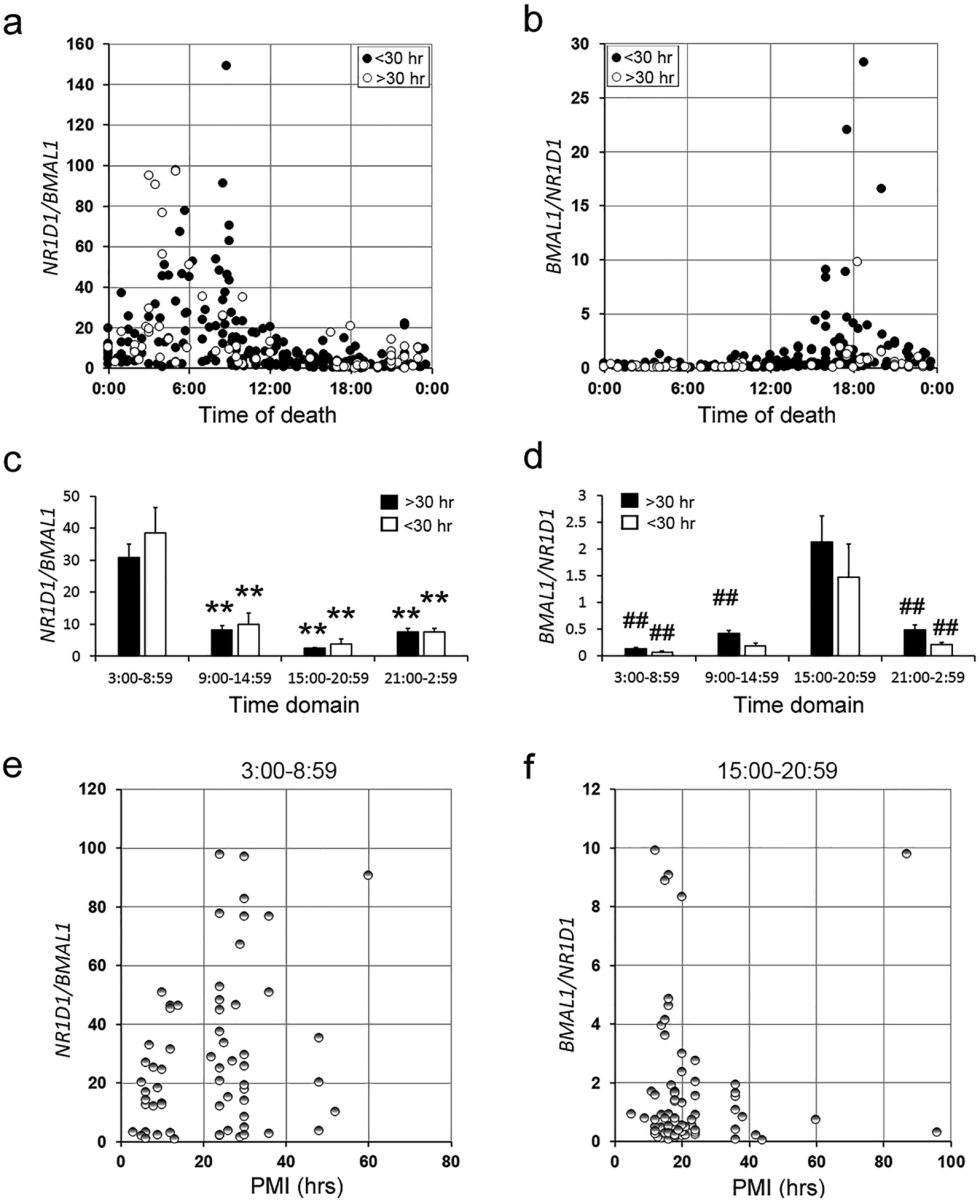
Assessment of the effect of postmortem interval on the *N/B* (**a**) and *B/N* (**b**) ratios in the hearts of the deceased. The autopsy cases were divided into two groups of postmortem interval, < 30 h (closed circles, n = 250) and > 30 h (open circles, n = 68). The *N/B* (**c**) and *B/N* (**d**) ratios in four-time domains were examined in the < 30 h (closed columns) and > 30 h (open columns) postmortem interval groups by multiple comparison tests. The *N/B* ratios in the 3:00–8:59 time domain (**e**) and the *B/N* ratios in the 15:00–20:59 time domain (**f**) were plotted against postmortem interval. **p < 0.01, 3:00–8:59 time domain versus other time domains; ^##^*p* < 0.01, 15:00–20:59 time domain versus other time domains.

Finally, we examined the effect of post-mortem intervals on the ratios. We divided the cases into two groups, < 30 h postmortem interval (n = 250) and > 30 h postmortem interval (n = 68). The *N/B* and *B/N* ratios in both groups showed peaks in the morning and evening, respectively, indicating that the post-mortem interval had virtually no effect on them (Fig. 4a–f). However, there was no significant difference in the *B/N* ratio between the evening and noon time domains in the > 30 h post-mortem interval group (Fig. 4d). This is likely due to the small number of cases (n = 9) in the noon time domain of the > 30 h post-mortem interval group. The *N/B* ratio in the morning and the *B/N* ratio in the evening were plotted against the postmortem interval; the results indicated that the ratios are independent of the postmortem interval (Fig. 4e and f).

### Evaluation of the cause of death affecting the biological clock

**Figure 5:**
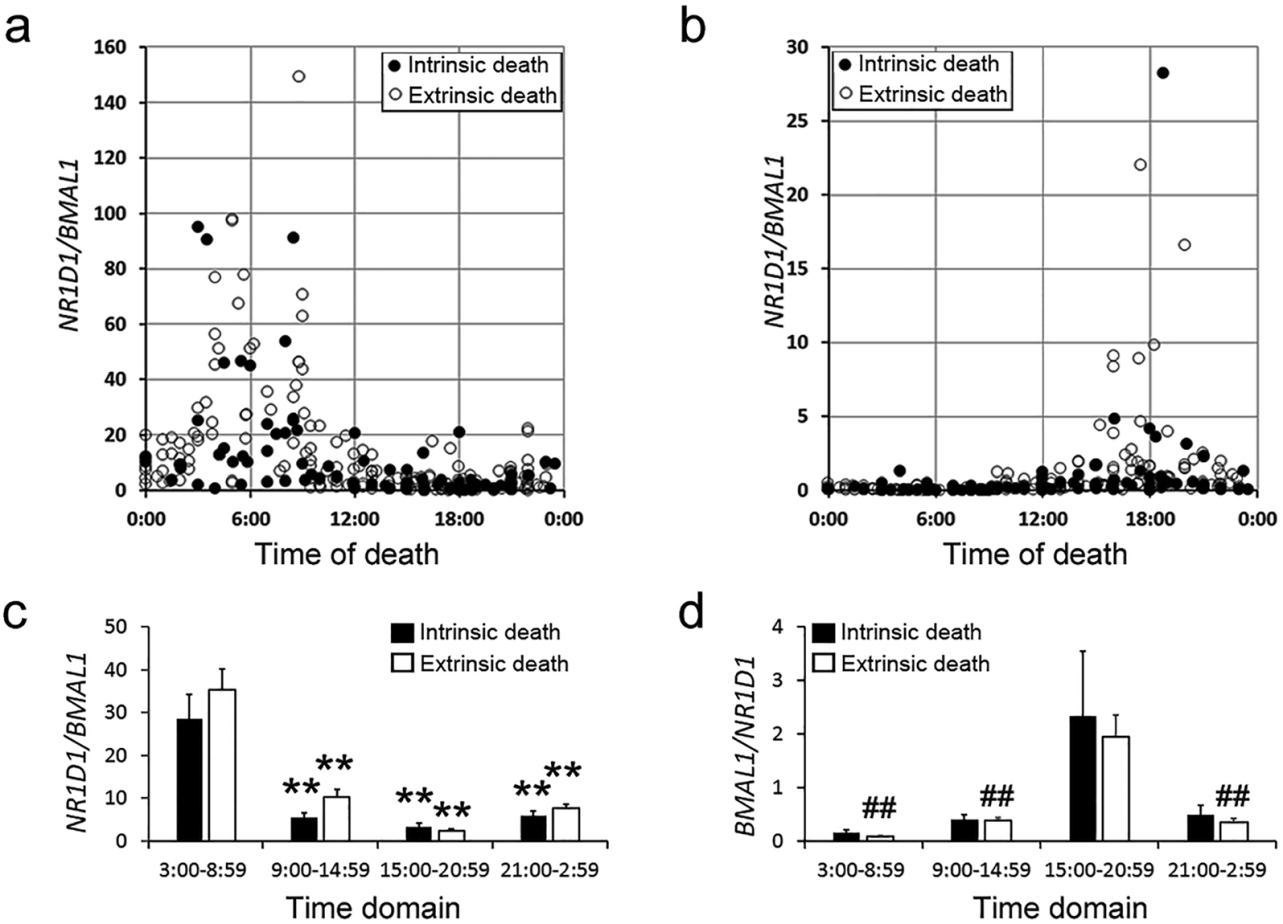
Assessment of the effect of the cause of death on the *N/B* (**a**) and *B/N* (**b**) ratios in the hearts of the deceased. The autopsy cases were divided into two groups, intrinsic death (closed circles, n = 73) and extrinsic death (open circles, n = 245). The *N/B* (**c**) and *B/N* (**d**) ratios in four-time domains were examined in the intrinsic death (closed column) and extrinsic death (open column) groups by multiple comparison tests. **p < 0.01, 3:00–8:59 time domain versus other time domains; ^##^*p* < 0.01, 15:00–20:59 time domain versus other time domains.

We next examined the differences in the temporal pattern of the *N/B* and *B/N* ratios between intrinsic (n = 73) and extrinsic (n = 245) death groups. As shown in Fig. 5a and b, there were no significant differences between the groups. In the extrinsic death cases, the *N/B* ratio in the morning and the *B/N* ratio in the evening were significantly higher than those in other time domains (Fig. 5c and d). However, in the intrinsic death cases, the *N/B* ratio in the morning was significantly higher than those in other time domains, but the *B/N* ratio in the evening did not significantly differ from those in other time domains (Fig. 5c and d). We also examined the effect of specific causes of death on the ratios. The most common causes of death (Table 1), including hemorrhagic and traumatic shock, aortic rupture, drowning, burn, asphyxia, intoxication, and ischemic heart failure, except brain injury, did not seem to have a significant effect on the ratios (data not shown).

**Table 1:** The autopsy cases examined.

	Number			age
	Total	Male	Female	
Extrinsic death (n = 245)				
Intoxication	14	7	7	23–91
Drowning	48	36	12	2–94
Hemorrhagic and traumatic shock	46	36	10	17–94
Asphyxia and hanging	29	18	11	6 m–84
Burn	33	25	8	30–97
Head injury	50	37	13	20–97
Others	25	17	8	18–92
Intrinsic death (n = 73)				
Ischemic heart failure and infarction	34	25	9	38–91
Circulation failure	18	10	8	5 m–58
Others	21	13	8	5–93

**Figure 6:**
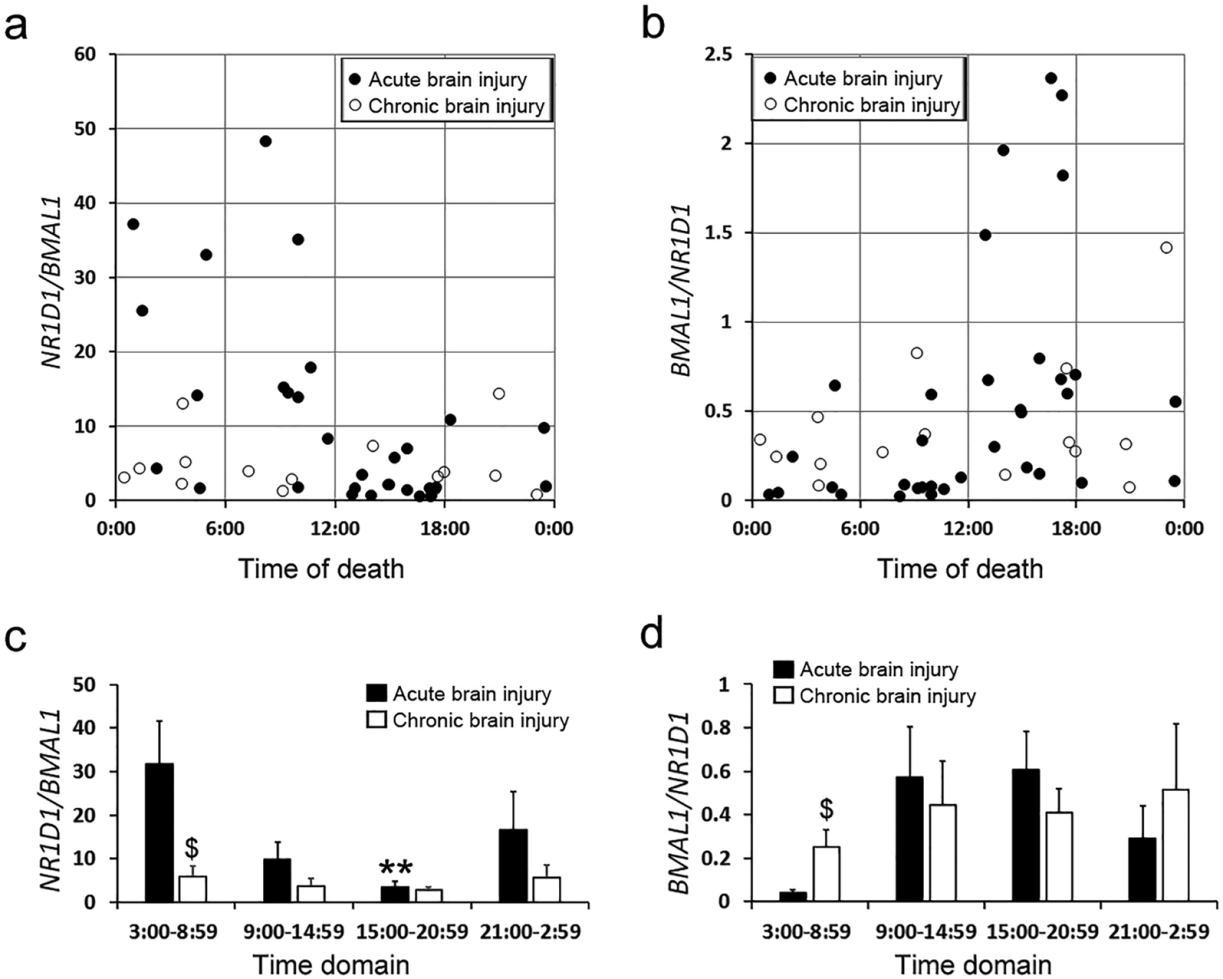
Assessment of the effect of severe brain injury on the *N/B* (**a**) and *B/N* (**b**) ratios in the hearts of the deceased. The severe brain injury cases were divided into two groups, cases of immediate death by acute brain injury (closed circles, n = 35) and those of protracted death by chronic brain injury with cerebral edema, cerebral hernia, or cerebral hypoxia (open circles, n = 15). The *N/B* (**c**) and *B/N* (**d**) ratios in four-time domains were examined in the immediate death by acute brain injury (closed columns) and protracted death by chronic brain injury (open columns) groups by multiple comparison tests. **p < 0.01, 3:00–8:59 time domain versus 15:00–20:59 time domain. ^$^*p* < 0.05, immediate death by acute brain injury versus protracted death by chronic brain injury.

Of note, brain injury, especially, chronic brain injury with cerebral edema, cerebral hernia, and cerebral hypoxia seemed to strongly affect the ratios in the hearts of the deceased. As shown in Fig. 6a and b, the morning peak of the *N/B* ratio and the evening peak of the *B/N* ratio did not take place in cases of delayed death due to chronic brain injury (n = 15), whereas the peaks of the *N/B* and *B/N* ratios were observed in acute death cases with severe brain injury (n = 35). The cases of delayed death due to chronic brain injury did not show an oscillation in the *N/B* and *B/N* ratios (Fig. 6c and d). The *N/B* ratio in the morning significantly differs from that in the evening in cases of acute death with severe brain injury (Fig. 6c). However, these findings are from a limited small number of cases, and the loss of oscillation of *N/B* and *B/N* ratios due to chronic brain injury needs to be confirmed in more cases.

### Applicability of our method to forensic practice

**Figure 7:**
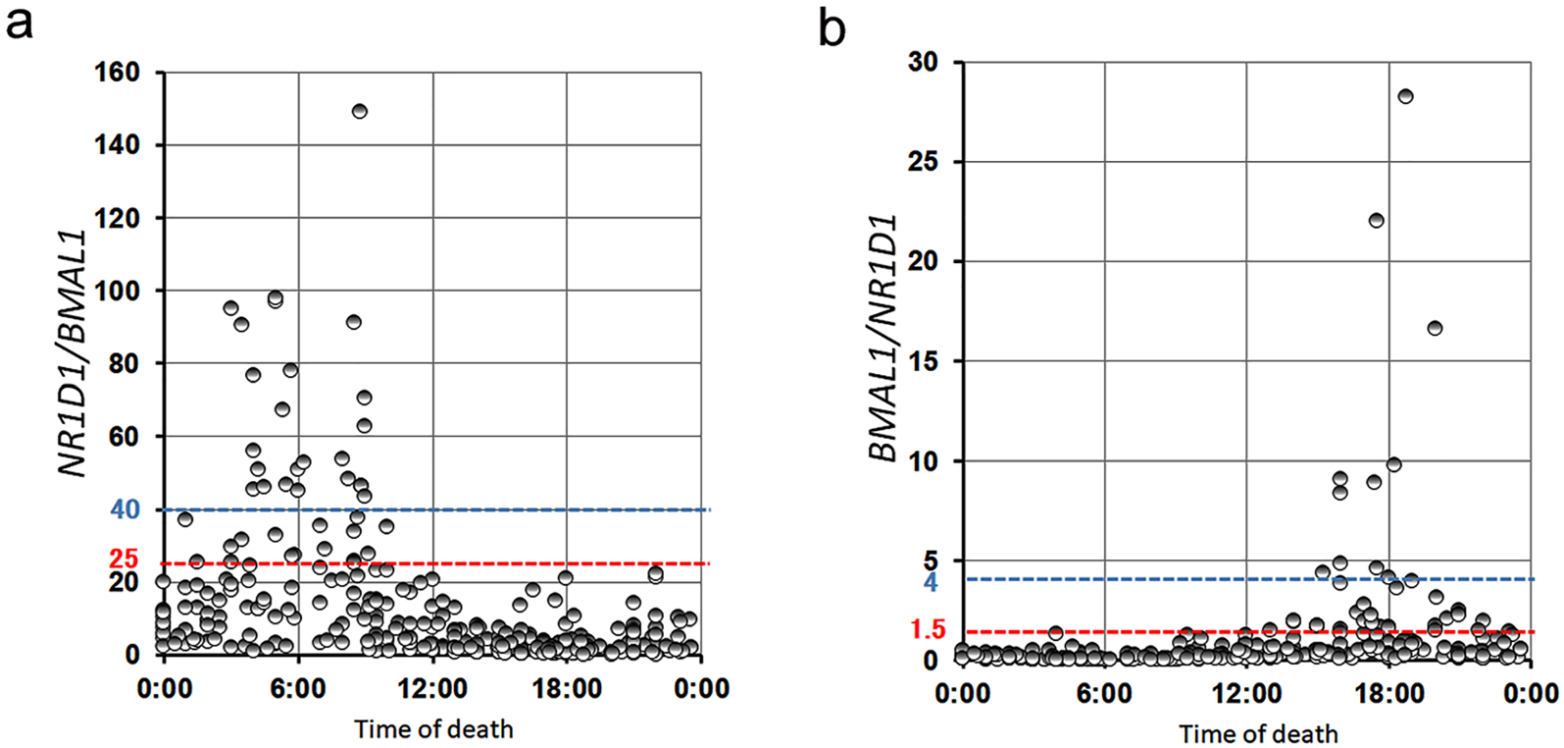
Criteria for applying our method to forensic practice. Temporal pattern of the *N/B* ratio in the heart of the deceased. (**a**) Cases with *N/B* ratio > 25 (red line) had a time of death between 1:00 and 10:00 (n = 40), and those with a ratio > 40 (blue line) had a time of death between 3:00 and 9:00 (n = 23). Temporal pattern of the *B/N* ratio in the hearts of the deceased. (**b**) Cases with *B/N* ratio > 1.5 (red line) had a time of death between 14:00 and 22:00 (n = 39), and those with a ratio > 4 (blue line) had a time of death between 15:00 and 20:00 (n = 11).

Our method reads the biological clock in the deceased; however, there are only two-time domains (morning, around 6:00; evening, around 18:00) in the clock. The *N/B* ratio is suitable for reading at 6:00 and the *B/N* ratio is suitable for reading at 18:00. All cases where the *N/B* ratio was > 25 were deaths occurring from 1:00 to 10:00 (n = 40), and those where the ratio was > 40 were deaths occurring from 3:00 to 9:00 (n = 23) (Fig. 7a). On the other hand, all cases where the *B/N* ratio was > 1.5 were deaths occurring from 14:00 to 22:00 (n = 39), and those where the ratio was > 4 were deaths occurring from 15:00 to 20:00 (n = 11) (Fig. 7b). However, only 24.8% (79/318) of morning and evening deaths were predicted by our method, and low values of N/B and B/N ratios do not exclude morning and evening deaths. Therefore, although this method is not effective in all cases, it is still important in forensic practice because it complements conventional methods from a completely different perspective.

## Discussion

To date, most methods for estimating the time of death estimate the time since death and are affected by internal, external, antemortem, and postmortem conditions. We hypothesized that the biological clock stops at the time of death, and developed a method to read this stopped biological clock^16^. Therefore, our method estimates the time of death, not the time since death, and appears to be independent of environmental factors; however, it can be influenced by internal factors such as age, gender, cause of death, and lifestyle of the deceased. The reliability and limitations of the practical application of newly developed methods must be evaluated. Thus, we examined our method in increased number of cases with a defined time of death. The *N/B* ratio showed a peak around 6:00, indicating that the method can give a stable result. Furthermore, we examined a novel reverse parameter, the *B/N* ratio, which showed a peak around 18:00. The *N/B* ratio was high in the morning, while the *B/N* ratio was high in the evening; therefore, we can determine whether death occurred in the morning or evening with this method. However, low *N/B* and *B/N* values were often found in cases of death at around 6:00 and 18:00, respectively. Such irregular values were not seen in the animal experiments because mice had a uniform genetic background and were bred in a strictly controlled environment^16^. Furthermore, all mice were sacrificed quickly by cervical dislocation under deep anesthesia. On the other hand, humans have different genetic backgrounds and live in various time patterns (e.g., shift workers), which might affect the expression pattern of biological clock genes^19,20^.

In the present study, we demonstrated that gender, age, and postmortem interval (within 96 h after death) did not significantly affect the *N/B* and *B/N* ratios. However, the youngest (< 1 year old, n = 5), and oldest (> 90 years old, n = 14) cases as well as those with long postmortem intervals (> 48 h, n = 11) were examined in a limited number. It is known that circadian rhythms such as body temperature and nocturnal sleep onset appear within 60 days after birth^21^. Moreover, the circadian oscillation of clock gene expression in the SNC (suprachiasmatic nucleus) and some peripheral tissues has been confirmed in nonhuman primate fetuses^21^, suggesting that clock gene expression in the heart of human infants may also show circadian oscillation. Therefore, the biological clock-based estimation of the time of death seems to be applicable to infant cases. However, maternal melatonin affects clock gene expression in nonhuman primate fetuses^22^, indicating that the breastfeeding pattern might affect the circadian clock in infants. Therefore, differences in clock gene expression patterns between the infant's and adult's heart may be found in future research. On the other hand, it has been reported that aging significantly affects the circadian pattern of gene expression in the human prefrontal cortex, which might bring about changes in the circadian rhythm in old age^23^. Different circadian rhythms in older individuals, especially the feeding pattern, can affect biological clock gene expression^19,20^. Since the biological clock in the peripheral tissues is also under adrenergic control^24^, age-related changes in the beta-adrenergic neuroeffector system might alter the clock gene expression pattern in the heart of older adults^25^. Based on the above-mentioned facts, our method should be applied carefully to infants and older adults. Longer postmortem intervals might cause RNA deterioration^26^, which increases the uncertainty of the results. Since the number of cases in children, the elderly, and cases with a long postmortem interval is small, a study using an increased number of cases is necessary for a statistically meaningful discussion.

The cause of death seemed to affect the *N/B* and *B/N* ratios. However, there were no significant differences in the temporal patterns between intrinsic and extrinsic death cases. Moreover, most causes of death did not significantly affect the ratios. Exceptionally, the peaks of both ratios almost disappeared in the cases of death with cerebral edema, cerebral hernia, or cerebral hypoxia. We also found an alteration of the *N/B* ratio in the iliopsoas muscle tissue of cases with chronic brain injury (not shown), suggesting that chronic brain injury-induced SCN damage brings about a systemic alteration of peripheral clock gene expression. Disturbances in circadian rhythms due to brain trauma have been reported^27,28,29^. Recently, traumatic brain injury-induced alteration of clock gene expression in the SCN and hippocampus was reported in a rat model^30^. Our preliminary result in mouse model of water intoxication showed that cerebral edema induced alteration of biological clock in the heart (Supplementary Data). Therefore, biological clock-based estimation of the time of death should be applied with caution to cases of severe brain injury or intrinsic death with diseases affecting brain function such as severe hepatic encephalopathy.

We analyzed 318 cases in the present study. However, there was bias in the number of cases with regards to gender, age, cause of death, and other factors. The number of cases in some groups, such as females, was less than 100, and some of these cases did not show statistical significance in the *N/B* and *B/N* ratios between morning and evening time domains compared to other time domains. Therefore, our method should be further validated with studies using a larger number of cases. Multifacility research may be necessary to conduct an analysis with a sufficient number of cases.

Recently, an analysis of human transcriptional rhythms using a cyclic ordering algorithm called Cyclops was reported^31^. The Cyclops algorithm enables the estimation of the circadian phase of a sample from high-throughput data that lack temporal information, and is expected to be an innovative approach to estimating the time of death in forensic practice. As Cyclops is an algorithm for the temporary reconstruction of population-based human organ data, its usefulness as a method for estimating the time of death for individual autopsy samples in forensic practice is uncertain. The usefulness and problems of Cyclops will be clarified by verifying it in forensic practice. Another problem is that high-throughput analysis is currently expensive for forensics.

In the present study, our method was able to predict only 79 cases of morning or evening deaths out of a total of 318 cases (about 25%). This indicates that our method only works in limited cases. However, all classical methods for estimating time of death have uncertainties, and are based on postmortem changes that begin at death and are influenced by various environmental factors. In contrast, our method directly estimates the death time based on the circadian clock, which stops at death and is unaffected by factors that influence postmortem changes. For example, after a deceased person's body temperature reaches ambient temperature, it is difficult to estimate time since death based on body temperature. In the case of burn death, many classical estimation methods, such as body temperature, corneal opacity and rigor mortis cannot be used. Therefore, all classical estimation methods have limitations in their applicability. Our method complements conventional methods from a completely different perspective and can be used where conventional methods are not applicable.

In conclusion, our method makes it possible to estimate the morning and evening deaths by reading the *N/B* and *B/N* ratios in the heart of the deceased, regardless of gender, age, postmortem interval, and cause of death. Although the *N/B* and *B/N* ratios cannot exclude the possibility of death occurring in the morning or evening, our method is still valuable in forensic practice because it can complement the classical methods that are dependent on postmortem changes. However, since severe brain injury profoundly affects the peripheral circadian clock, our method may not apply to cases of severe brain injury. Additionally, the applicability of the method to infants and older adults needs to be evaluated in more cases.

## Methods

### Autopsy samples

Heart samples were obtained from 318 forensic autopsy cases with known times of death (224 men and 94 women). The age of autopsied subjects ranged from 2 months to 97 years (average: 58.7 years), and postmortem intervals in all cases were less than 96 h (average: 22.3 h). The causes of death of the subjects were shown in Table 1. Tissue samples were taken during autopsy, immediately frozen in liquid nitrogen and stored at − 80 °C until use. Clock gene expression is routinely analyzed in all autopsy cases at our Institute as part of the process for estimating the time of death.

### Extraction of total RNA and real-time RT-PCR

Total RNA was extracted from tissue samples (about 100 mg) and applied to Maxwell System with Maxwell RSC simplyRNA Tissue Kit (Promega Corporation, Madison, WI) according to the manufacturer’s instructions. Then 1 μg of total RNA was reverse-transcribed into cDNA by using a PrimeScript RT reagent Kit (TAKARA BIO INC., Otsu, Japan) with six random primers (TAKARA BIO INC.). Thereafter, generated cDNA was subjected to qPCR analysis using a SYBR^®^ *Premix Ex Taq*™ II kit (TAKARA BIO INC.) with specific primer sets (Table 2). Amplification and detection of mRNA were performed using Thermal Cycler Dice^®^ Real Time System (TP800, TAKARA BIO INC).

**Table 2:** Sequences of the primers used for real-time PCR.

Transcript	Sequence^a^
*BMAL1*	(F) 5′-GCCTACTATCAGGCCAGGCTCA-3′
	(R) 5′-AGCCATTGCTGCCTCATCATTAC-3′
*NR1D1*	(F) 5′-TCAGCTGGTGAAGACATGACGAC-3′
	(R) 5′-GGAGCCACTGGAGCCAATGTA-3′

^a^(F) Forward primer; (R) Reverse primer.

### Statistical analysis

Data were expressed as the mean ± standard error of the mean. Unpaired Student *t*-test and Scheffe’s F test were performed to compare the values between two groups and for multiple comparisons, respectively. Statistical significance was set at *p* < 0.05.

### Ethical approval

Our study was approved by the Research Ethics Committee of Wakayama Medical University (No. 3177). All procedures were carried out in accordance with the principles of the Declaration of Helsinki. In addition, this study was conducted using past autopsy records and heart tissues; we were unable to obtain informed consent from the bereaved family for the use of the records and the heart tissues. In accordance with the "Ethical Guidelines for Medical Research Involving Human Subjects (enacted by the Ministry of Health, Labor and Welfare in Japan)," Sect. 12–1 (2) (a) (c), the review board of the Research Ethics Committee of Wakayama Medical University waived the need for written informed consent from relatives of the individuals studied because this was a de-identified retrospective study of archived autopsy-derived tissues.

## Supplementary Information

Supplementary Information.

## Data Availability

The authors declare that all data are available upon request. All requests should be made to Dr. Toshikazu Kondo.
