# Supplementary material for: Application and limitation of a biological clock-based method for estimating time of death in forensic practices
Source: Sci Rep. 2023 Apr 13;13:6093. doi: 10.1038/s41598-023-33328-3 (PMC10102023; doi:10.1038/s41598-023-33328-3)
Supplement: Supplementary file 1 — Supplementary Information. [file 41598_2023_33328_MOESM1_ESM.docx]

Supplementary data, Kimura at. A

**Application and limitation of a biological clock-based method for estimating time of death in forensic practices**

**Akihiko Kimura^1)^, Yuko Ishida^1)^, Mizuho Nosaka^1)^, Akiko Ishigami^1)^, Hiroki Yamamoto^1)^, Yumi Kuninaka^1)^, Satoshi Hata^2)^, Mitsunori Ozaki^3)^, Toshikazu Kondo*^1)^**

1. Department of Forensic Medicine, Wakayama Medical University
2. [Department of Cardiovascular medicine, Kinan Hospital](https://minamiwakayama.hosp.go.jp/)
3. Department of Neurological surgery, National Hospital Organization Minami Wakayama Medical Center

Corresponding author: Toshikazu Kondo, MD & PhD, Department of Forensic Medicine, Wakayama Medical University, 811-1 Kimiidera, Wakayama 641-8509, Japan

Tel. & Fax: +81-73-441-0641, E-mail: [kondot@wakayama-med.ac.jp](mailto:kondot@wakayama-med.ac.jp)

**Effects of water-intoxication-induced cerebral edema on the cardiac biological clock in mouse**


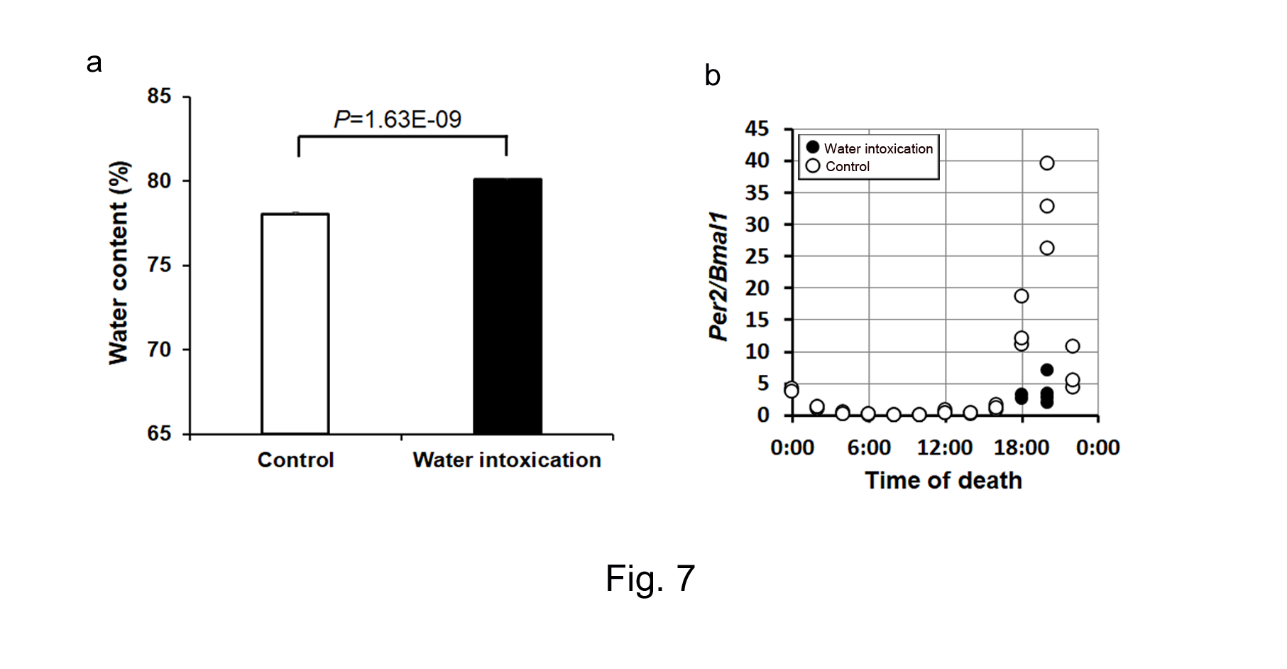
Cerebral edema strongly affected the biological clock in the heart of cases with severe brain injury. Therefore, we examined the effects of water intoxication-induced cerebral edema on clock gene expression in mouse hearts. Intraperitoneal administration of a large water mass (19% of the body weight) containing desmopressin (1) induced severe cerebral edema (Fig. 7a). In this experiment *Per2/Bmal1* ratio was analyzed instead of *Nr1d1/Bmal1* ratio. *Per2* is one of same clock gene as *Nr1d1* and is a molecule responsible for the control mechanism of the biological clock. The circadian expression of Nr1d1 and Per2 is essentially controlled by the same molecular mechanism. Two hours after water administration, mice went into a coma, and the *Per2*/*Bmal1* ratio in the hearts was significantly reduced compared with that in the hearts of control mice (Supplemental Fig. 1), suggesting that severe cerebral edema-induced malfunction of the central clock in the suprachiasmatic nucleus (SCN) alters clock gene expression in the heart.

Supplementary figure 1. Water intoxication-induced cerebral edema and alteration of clock gene expression in mouse hearts. Whole brain water content in control (open columns) (n = 6) and mass water-administered (closed columns) mice (n = 6). (a) The *Per2/Bmal1* ratio in the hearts of intoxicated mice collected around 18:00 (n = 2) and 20:00 (n = 4) (closed circles). (b) The *Per2/Bmal1* ratio in control mouse hearts collected at 2 h intervals (n = 3 at each time point) (open circles).

**Reference**

1. Manley GT, Fujimura M, Ma T, Noshita N, Filiz F, Bollen AW, Chan P, Verkman AS. [Aquaporin-4 deletion in mice reduces brain edema after acute water intoxication and ischemic stroke.](https://pubmed.ncbi.nlm.nih.gov/10655103/) Nat Med. 2000; 6:159-63. doi: 10.1038/72256.

**Methods**

*Mouse model of water intoxication-induced cerebral edema*

Specific pathogen-free 8-10-week-old male mice (C57BL/6J) were obtained from Japan SLC (Hamamatsu, Japan). All mice were bred and housed at a constant temperature (23 ± 2°C), with a 12 h light/dark cycle (light on at 08:00 h and off at 20:00 h). They were fed with standard feed and given water *ad libitum*. Mice (n=6) were intraperitoneally administrated with distilled water (19% body weight) containing Desmopressin (0.4 mg/kg body weight) obtained from FUJIFILM Wako Pure Chemical (Osaka, Japan) (19). Two hours after water administration, mice were sacrificed by cervical dislocation under deep Isoflurane anesthesia, and the brain and heart were collected. The brain was also collected from control mice (n=6) as described above. Hearts were immediately stored −80°C until RNA extraction. *Per2* and *Bmal1* expression in the hearts was analyzed by RT-qPCR. Whole brains were subjected to water content measurement. Percent brain water content was calculated by the following equation: 100 x (wet weight − dry weight)/wet weight. For analysis of clock genes circadian oscillation in control mice, mice (n=3/time point) were sacrificed at 2 hr intervals, and the hearts were collected as described above. All animal experiments were approved by the Committee on Animal Care and Use of Wakayama Medical University (No. 730) and complied with the ARRIVE guidelines. All experiments were performed in accordance with relevant guidelines and regulations.

**Supplemental table 1. Sequences of the primers used for real-time PCR**

| **Transcript** | **Sequence**^a^ |
| --- | --- |
| *Bmal1*  *Per2* | (F) 5’-TCAGATGACGAACTGAAACACCTAA-3’  (R) 5’-TTTGGATGCAGGTAGTCAAACAAG-3’  (F) 5’-ATCAGCCATGTTGCCGTGTC-3’  (R) 5’-CGTGCTCAGTGGCTGCTTTC-3’ |

^a^ (F) Forward primer; (R) Reverse primer
